# Synthesis and structure–activity relationships of 2- and/or 4-halogenated 13***β***- and 13α-estrone derivatives as enzyme inhibitors of estrogen biosynthesis

**DOI:** 10.1080/14756366.2018.1490731

**Published:** 2018-09-19

**Authors:** Ildikó Bacsa, Bianka Edina Herman, Rebeka Jójárt, Kevin Stefán Herman, János Wölfling, Gyula Schneider, Mónika Varga, Csaba Tömböly, Tea Lanišnik Rižner, Mihály Szécsi, Erzsébet Mernyák

**Affiliations:** a Department of Organic Chemistry, University of Szeged, Szeged, Hungary;; b 1st Department of Medicine, University of Szeged, Szeged, Hungary;; c Department of Microbiology, University of Szeged, University of Szeged, Szeged, Hungary;; d Laboratory of Chemical Biology, Institute of Biochemistry, Biological Research Centre of the Hungarian Academy of Sciences, Szeged, Hungary; e Institute of Biochemistry, Faculty of Medicine, University of Ljubljana, Ljubljana, Slovenia

**Keywords:** Estrone, halogenations, aromatase, STS, 17β-HSD1

## Abstract

Ring A halogenated 13α-, 13β-, and 17-deoxy-13α-estrone derivatives were synthesised with N-halosuccinimides as electrophile triggers. Substitutions occurred at positions C-2 and/or C-4. The potential inhibitory action of the halogenated estrones on human aromatase, steroid sulfatase, or 17β-hydroxysteroid dehydrogenase 1 activity was investigated via *in vitro* radiosubstrate incubation. Potent submicromolar or low micromolar inhibitors were identified with occasional dual or multiple inhibitory properties. Valuable structure–activity relationships were established from the comparison of the inhibitory data obtained. Kinetic experiments performed with selected compounds revealed competitive reversible inhibition mechanisms against 17β-hydroxysteroid dehydrogenase 1 and competitive irreversible manner in the inhibition of the steroid sulfatase enzyme.

## Graphical Abstract

**Figure:**
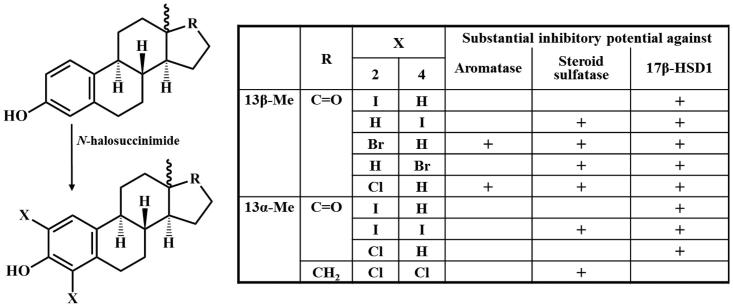

## Introduction

**Scheme 1.**
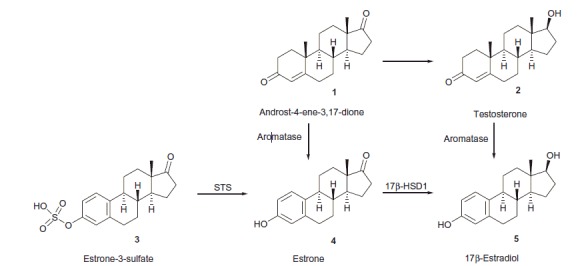
The role of aromatase, STS and 17β-HSD1 enzymes in the estrogen biosynthesis.

Estrogens play an important role in cell proliferation and their overproduction stimulates the growth of hormone-sensitive cells, leading to hormone-dependent diseases, such as breast and endometrial cancer^1^. Inhibition of enzymes involved in the final steps of estrogen biosynthesis is a powerful route to prevent the proliferative action of estrogens. Cytochrome P450 aromatase is responsible for the conversion of nonaromatic androgens **1** and **2** to estrone (E1, **4**, Scheme 1) or 17β-estradiol (E2, **5**), respectively. Estrogens are originated not only from nonaromatic steroids, but also from estrone-3-sulfate (E1S) **3**, which exists as a large circulatory reservoir. E1S is transported into cells by organic anion transporters (OATPs) and several other members of the SoLute Carrier (SLC) protein family^2^. After entering the cells, E1 is released from the sulfate ester by steroid sulfatase (STS). The next, hormone-activating process is the formation of active hormone E2 from E1, which is mainly catalyzed by 17β-hydroxysteroid dehydrogenase type 1 (17β-HSD1). Activities of STS and 17β-HSD1 are higher in breast cancer tissue compared to other tissues, that is this may be the main route of local estrogen production in the tumors^3^ ^,^ ^4^.

**Figure 1.**
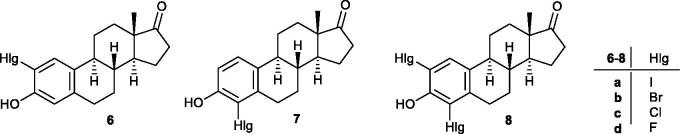
Ring A halogenated derivatives **6–8** of estrone.

Inhibition of the above-mentioned enzymes may be achieved by inhibitors designed on the estrane core. A-ring halogenation of E1 results in 2- and 4-regioisomeric (**6**, **7**) and/or 2,4-*bis*-substituted compounds (**8**, Figure 1)^5–7^. The substitution pattern of the aromatic ring and the nature of the introduced halogen greatly influence the inhibitory properties of estrone derivatives halogenated at the A-ring. Compounds obtained by introduction of the same substituent to different positions of the aromatic ring may possess high binding affinities to different enzymes. 2-Bromo- (**6b**) or 2-chloroestrone (**6c**) are potent aromatase inhibitors with low micromolar IC_50_ values^5^. Their 4-substituted counterparts (**7b**,**c**) display only moderate aromatase inhibitory potential. Möller *et al*. described that 2-haloestrones (**6b**,**c**) as 17β-HSD1 inhibitors can suppress the E1–E2 conversion with IC_50_ values in the submicromolar range^6^. 4-Halogenated counterparts (**7**) have not been tested against 17β-HSD1 by this research group. However, the 4-haloestrones (**7b**,**d**) are known to be efficient STS inhibitors^7^. Thus, introduction of Br or F onto C-4 of E1 led to a significant increase in STS inhibitory potential. None of the mentioned references discusses the affinity of 2-iodo- and 4-iodoestrones (**6a**, **7a**) or 2,4-*bis*-compounds (**8**) for these three enzymes.

Concerning the results obtained so far for certain A-ring halogenated estrones it seems that substitution of E1 at C-2 may enhance aromatase and 17β-HSD1 inhibitory potential, but 4-halogenation may lead to efficient STS inhibitors.

**Scheme 2.**
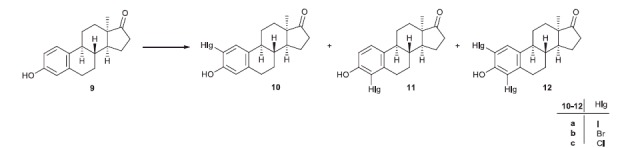
Ring A substitutions in the 13α-estrone series.

The use of the estrone-based inhibitors of the mentioned steroidogenic enzymes in the therapy is limited because of their retained estrogenic activity. The availability of inhibitors acting selectively without hormonal behavior would be of particular interest. Literature data reveal that estrogenic effect of estrone is a 5–35% extent of that of 17β-estradiol^8–10^. Chloro substitution at position C-4 of estrone or 17β-estradiol retains the estrogenicity, however, 4-bromo and 4-iodo, as well as 2-chloro, -bromo, and -iodo compounds exert suppressed effect compared to that of the parent compounds. Data vary according to the methods applied, but it can be stated that estrogenic effect decreases with the increasing size of the introduced halogen. C-2 substituted estrone or 17β-estradiol compounds are usually less estrogenic than their C-4 counterparts. The 2,4-disubstituted analogs, nevertheless, exert negligible estrogenic potential^8^ ^,^ ^10^ ^,^ ^11^. Certain other chemical modifications of the estrane skeleton, such as the inversion of the configuration at C-13 or the opening of ring D, may result in the complete loss of hormonal activity^12–15^. We have promising preliminary results concerning the design, synthesis and biochemical evaluation of 17β-HSD1 inhibitors based on hormonally inactive 13α-estrane core^16^. 13α-Estrone (**9**, Scheme 2) itself was proved to be a potent inhibitor with an IC_50_ comparable to that of the natural substrate E1. Additionally, the previously synthesised 13α-estrone derivatives (**10a**,**b**–**12a**,**b**) brominated or iodinated in the A-ring exerted low micromolar or submicromolar inhibitory potential^17^. Chlorinations in the 13α-estrone series leading to **10c**–**12c** have not been performed up to now. Concerning recent promising results, that 13α-estrone derivatives might possess valuable 17β-HSD1 inhibitory potential, it seemed rational to evaluate these compounds as potential aromatase or STS inhibitors, too.

**Scheme 3.**
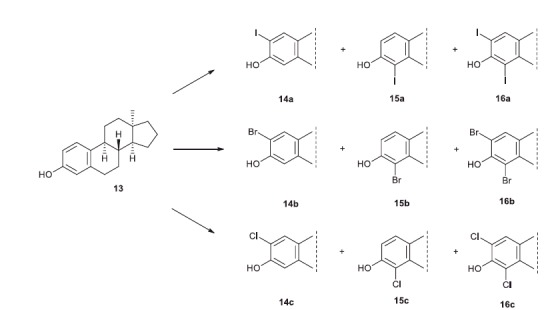
Introduction of different halogens onto the aromatic ring of 17-deoxy-13α-estrone

Based on the above-mentioned literature results here we aimed to perform aromatic halogenations on 13α-estrone (**9**) and its 17-deoxy counterpart (**13**, Scheme 2, 3). Chlorination of 13α-estrone (**9**) and introduction of chlorine, bromine or iodine onto positions 2 and/or 4 of 17-deoxy-13α-estrone (**13**) were planned. Synthesis of the halogenated 13β-estrone derivatives (**6a**,**b**,**c**–**8a**,**b**,**c**) was also designed with the aim of preparing the 13β-counterparts for comparative investigations (Figure 1). We additionally intended to investigate the potential *in vitro* inhibitory effects of the basic and the halogenated compounds (**6a**,**b**,**c**–**8a**,**b**,**c**; **9**–**16**) towards aromatase, STS and 17β-HSD1 enzymes. Comparison of the inhibitory results and evaluation of structure–activity relationships were also planned. Representatives from the most effective test compounds were selected for mechanistic and kinetic investigations.

## Materials and methods

Chemical syntheses and characterisation data of the reported compounds, as well as experimental conditions of enzymatic assays performed are described in the Supporting Information.

## Results and discussion

### Chemistry

We recently described the halogenations of the aromatic ring of **9** with different groups at position 3 (OH, OMe, OBn)^17^. Bromination or iodination was carried out with *N*-halosuccinimides as electrophile triggers in different solvents. In the 3-OH series both 2- and 4-substituted regioisomers were formed and *bis*-substitutions also occurred. The mixtures of the desired products (**10a**,**b**–**12a**,**b**) could efficiently be separated by flash chromatography.

As a completion of our previous results, here we performed the chlorination of the aromatic ring of **9** in order to get the appropriate 2-, 4-, or 2,4-*bis*-chloro derivatives (**10c**–**12c**) for further structure–activity examinations. Reactions with *N*-chlorosuccinimide led to a mixture of products (**10c**–**12c**, Scheme 2, Table 1, Entries 1, 2), which were separated by flash chromatography.

**Table 1.** Synthesis of halogenated compounds (**6–8, 10c–12c, 14–16**)

Entry	Starting compound	Electrophilic reagent (solvent)	Product	Yield (%)
1	**9**	NCS (1.0 equiv., TFA)	**10c** + **11c**	16 + 63
2	**9**	NCS (2.0 equiv., TFA)	**11c** + **12c**	57 + 19
3	**13**	NIS (1.5 equiv., TFA)	**14a** + **15a** + **16a**	48 + 24 + 24
4	**13**	NBS (1.1 equiv., CH_2_Cl_2_)	**14b** + **15b** + **16b**	44 + 33 + 11
5	**13**	NCS (1.5 equiv., CH_3_CN)	**14c** + **15c**	48 + 24
6	**13**	NCS (3.0 equiv., CH_3_CN)	**15c** + **16c**	26 + 53
7	**4**	NIS (1.0 equiv., TFA)	**6a** + **7a**	58 + 30
8	**4**	NIS (2.0 equiv., TFA)	**7a** + **8a**	59 + 30
9	**4**	NBS (1.0 equiv., CH_2_Cl_2_)	**6b** + **7b**	40 + 41
10	**4**	NBS (2.0 equiv., CH_2_Cl_2_)	**7b** + **8b**	12 + 72
11	**4**	NCS (1.1 equiv., MeCN/TFA)	**6c** + **7c**	30 + 45
12	**4**	NCS (2.2 equiv., CH_2_Cl_2_)	**7c** + **8c**	41 + 40

Expecting to acquire valuable structure–activity relationship data, it seemed reasonable to synthesise the appropriate halogenated 13α compounds in the 17-deoxy series, too. The halogenations of 17-deoxy-13α-estrone **13** were carried out using *N*-halosuccinimides in different solvents (Scheme 3, Table 1). Iodination of **13** with 1.5 equivalent of NIS in trifluoroacetic acid yielded the mixture of the two regioisomers (**14a** and **15a**) and the *bis*-compound (**16a**) in a ratio of 2:1:1 (Table 1, Entry 3). Bromination of **13** with 1.1 equivalent of reagent in dichloromethane led to a 4:3:1 mixture of **14b**, **15b** and **16b** (Table 1, Entry 4). Chlorination of **13** with 1.5 equivalent of NCS yielded the 2:1 mixture of the two regioisomers **14c** and **15c** (Table 1, Entry 5). The *bis*-chloro compound (**16c**) could be synthesised by using 3.0 equivalent of NCS (Table 1, Entry 6).

In order to have the appropriate 13β-estrone derivatives (**6a**,**b**,**c**–**8a**,**b**,**c**) for the comparative enzyme inhibition studies, halogenations were carried out in this series, too. Our primary goal was to get the two regioisomers and the *bis*-compounds for the *in vitro* tests; therefore the regioselectivity was inessential. Different conditions were needed for the convenient synthesis of mono- and disubstituted 13β compounds. Monosubstitutions occurred using nearly 1 equiv. of *N*-halosuccinimide (Table 1, Entries 7, 9, 11), but the excess (nearly 2 equiv.) of the halogenating agent led to disubstitution (Table 1, Entries 8, 10, 12), too. The aimed halogenations at C-2 and/or C-4 were achieved and the flash chromatographic separations of the reaction mixtures furnished the desired compounds (**6**–**8**).

### Enzyme inhibition studies

Aromatase, STS and 17β-HSD1 belong to different enzyme families with distinct catalytic mechanisms^18–20^. Specific inhibitors of these enzymes may be developed because of the differences in their active sites^4^. Dual or multiple inhibition might also be of value since inhibition of only one of these three enzymes is not adequate in treatment of hormone-dependent breast cancer. Since aromatase is needed for the synthesis of E1, hormone-dependent breast cancer may be more effectively treated by dual inhibition of aromatase and STS. Among the inhibitors of the mentioned three enzymes, aromatase inhibitors are clinically the most effective for hormone-dependent breast cancer.

Beside their efficiency, the estrogen deprivation is accompanied with resistance and side effects. It would be crucial to design new drug candidates without such side effects. Innovative treatment strategies combining inhibitors of STS or 17β-HSD1 with aromatase inhibitor could lower the dose of the latter and extend the disease progression. The tumor-selective lowering of E2 levels could be achieved by the use of inhibitors of these two enzymes together with the aromatase inhibitor.

Literature data reveal that three-dimensional structures of these enzymes have not met expectations in drug design, but they are useful in understanding the catalytic mechanisms and inhibitor binding^4^. Aside from the differences in the active sites and catalytic mechanisms, these three enzymes may be inhibited by similar, estrone-based inhibitors. Slight differences in the structures of the potential inhibitors, involving regioisomerism, may influence not only the extent but also the nature of inhibition. This seems to be true for the ring A halogenated derivatives in the 13β-estrone series^5–7^. Literature data suggest that substitutions at C-2 are advantageous concerning 17β-HSD1 and aromatase, but 4-regioisomers are better STS inhibitors. Nevertheless, there is no thorough comparative investigation in the literature concerning the aromatase, STS and 17β-HSD1 inhibitory activities of 2-, 4-, and 2,4-*bis*-chloro, -bromo, and -iodo estrones. The literature data are incomplete in this sense. The involvement of 17-keto- and 17-deoxy-13α-estrone compounds in the structure–activity determinations seems also reasonable. To the best of our knowledge, there has not been found any exact correlation between the structural characteristics of the investigated estrone derivative (regarding the conformation and the presence of the 17-keto function) and good aromatase, STS, or 17β-HSD1 inhibitory potential.

As a part of our efforts to get valuable pieces of information, which could help the development of more potent single or multiple inhibitors of estrogen biosynthesis, we describe here the evaluation of halogenated 17-keto-13β-, 17-keto-13α-, and 17-deoxy-13α-estrone derivatives (**6a**,**b**,**c**–**8a**,**b**,**c**, **9**–**16**) as 17β-HSD1, aromatase, and/or STS inhibitors.

Enzyme inhibition studies were performed by *in vitro* radiosubstrate incubations using human term placenta cytosol and microsomas as enzyme sources. Aromatase inhibition was measured on testosterone (**2**) to E2 (**5**) conversion, STS inhibition was investigated via hydrolytic release of E1 (**4**) from E1S (**3**), whereas the influence on 17β-HSD1 was tested by the transformation of (**4**) to E2 (**5**). Relative conversions compared to non-inhibited controls (100%) were measured in the presence of 10 µM concentration of the test compound. For more efficient compounds, IC_50_ values were determined and inhibitory potentials were assessed also in comparison to IC_50_ data of the corresponding substrate. Reference IC_50_ parameters measured for the substrates and the basic compound E1 (**4**) are listed in Table 2. Mechanistic and kinetic investigations were performed and inhibitory constants (*K_i_*) were determined for selected compounds.

**Table 2.** Reference IC50 parameters of the substrate compounds. Relative conversions (Rel. conv., control incubation with no inhibition is 100%) measured in the presence of 10 μM concentration of the compound tested. Mean ± SD, n = 3.

Comp.	Structure	Arom	STS	17β-HSD1
		IC_50_ ± SD (µM)	IC_50_ ± SD (µM)	IC_50_ ± SD (µM)
Testosterone (**2**)		0.52 ± 0.14		
E1S (**3**)			5.2 ± 1.2	
E1 (**4**)		>10 Rel. conv. 78 ± 7%	24 ± 10	0.63 ± 0.11

IC_50_: the inhibitor concentration decreasing the enzyme activity to 50%. *K_i_*: inhibitor constant determined from Dixon plot; SD: standard deviation.

### Aromatase

According to literature information, introduction of F, Cl, Br, Me, and formyl groups to C-2 of E1 affords compounds with high binding affinity to aromatase enzyme^5^. 2-Bromoestrone (**6b**) proved to be an efficient inhibitor with an IC_50_ value of 2.4 µM, whereas 2-chloroestrone (**6c**, *K_i_* = 0.13 µM) seemed to be more potent displaying about a 20-fold enhancement in its affinity compared to E1 (*K_i_* = 2.50 µM). Concerning **6c** as a powerful competitive inhibitor, no evidence of enzymatic generation of a reactive substance was observed. Exact correlations between inhibitory activity and size and/or electronegativity of substituents at C-2 could not be established. Inhibitory activities of estrone analogs were found to be higher than those of the corresponding estradiol derivatives. Consequently, a 17-carbonyl function plays a crucial role in the binding of estrogens to the active site of aromatase enzyme, as observed in the cases of the androgen derivatives^21–24^. Halogenation at the C-4 position, except for fluorination, markedly decreased affinities. Osawa *et al*. suggested that substrates bind to the active site of aromatase through two conformations [β-side up (normal) and α-side up (upside down)], or have the opportunity and space to rotate around the binding site^25^. In the β-side up binding mode, C-2 is located close to the heme. This binding allows the catalysis of 2-hydroxylation. The estradiol molecule may rotate by 180° through the O(3)–O(17) axis, resulting in the α-side up binding mode, which allows 4-hydroxylation but to a lesser extent. The higher inhibitory potential of C-2-substituted E1 analogs compared with those of their C-4 substituted counterparts may be related to the high aromatase C-2 hydroxylation activities. These literature results indicate that concerning estrone-based aromatase inhibitors, the nature of the C-17 substituent, the substitution pattern of the aromatic ring, and the conformation of the compound greatly influence their inhibitory behavior.

In this contribution we also report *in vitro* aromatase inhibition tests of the synthesised 13β- and 13α-estrone derivatives. Certain 2-halogenated 13β-estrone derivatives (**6b** and **6c**) displayed low micromolar inhibition (Table 3). 2-Chloroestrone (**6c**) was found to be the most effective with its IC_50_ value of 6.0 µM. 2-Bromoestrone (**6b**) was slightly less potent (IC_50_ = 8.7 µM). These results are in a good agreement with those of Numazawa *et al*.^5^. 2-Iodoestrone (**6a**) displayed weaker inhibition with a relative conversion of 66% at a 10 µM test concentration (IC_50_ > 10 µM). Nevertheless, both derivatives have enhanced efficiency compared to their parent compound E1. The results obtained for the 2-halogenated 13β-estrone derivatives reveal that the inhibitory potential decreases with the increasing size of the halogen substituent.

**Table 3.** *In vitro* inhibition of enzyme activities by the test compounds.

		Arom		STS		17β-HSD1	
Comp.	Structure	Rel.conv. ± SD (%)	IC_50_ ± SD (µM)	Rel.conv. ± SD (%)	IC_50_ ± SD (µM)	Rel.conv. ± SD (%)	IC_50_ ± SD (µM)
				
							
**6a**		62 ± 1		64 ± 3			0.064 ± 0.034
**7a**		88 ± 2			0.23 ± 0.09 *K_i_* = 0.36 ± 0.05 μM		0.36 ± 0.25
**8a**		86 ± 6		80 ± 13		55 ± 7	
**6b**			8.7 ± 2.8		2.0 ± 0.4		0.095 ± 0.031
**7b**		91 ± 6			0.89 ± 0.3		0.30 ± 0.20
**8b**		81 ± 5			2.1 ± 0.6		0.96 ± 0.45
**6c**			6.0 ± 1.2		2.4 ± 0.4		0.18 ± 0.02
**7c**		92 ± 3			1.6 ± 0.3		0.60 ± 0.16
**8c**		82 ± 4			3.0 ± 0.9		0.59 ± 0.16
							
				
							
**9**		85 ± 13		96 ± 1			1.2 ± 0.2 [12] *K_i_* = 1.9 ± 0.2 μM
**10a**		82 ± 10		83 ± 3			0.59 ± 0.23 [13]
**11a**		90 ± 7			6.0 ± 1.6		1.0 ± 0.3 [13] *K_i_* = 2.2 ± 0.3 μM
**12a**		91 ± 1			2.4 ± 0.5		0.38 ± 0.08 [13] *K_i_*= 0.94 ± 0.15 μM
**10b**		100 ± 5		81 ± 6			1.2 ± 0.3 [13]
**11b**		78 ± 1			8.5 ± 3.1		2.1 ± 1.2 [13]
**12b**		108 ± 11		71 ± 4			2.7 ± 0.1 [13]
**10c**		106 ± 6		57 ± 1			0.33 ± 0.10
**11c**		98 ± 3		80 ± 7			2.6 ± 1.0
**12c**		101 ± 4		70 ± 3			2.2 ± 0.6
							
				
							
**13**		95 ± 12		76 ± 5			1.1 ± 0.33 *K_i_* = 2.0 ± 0.4μM
**14a**		89 ± 9			3.9 ± 1.6		2.9 ± 1.6
**15a**		92 ± 7			2.7 ± 1.3	57 ± 9	
**16a**		94 ± 2		59 ± 13		61 ± 7	
**14b**		97 ± 5			4.1 ± 1.3		1.3 ± 0.8
**15b**		90 ± 8			3.7 ± 1.2	49 ± 12	11 ± 4
**16b**		82 ± 6			7.5 ± 2.0		4.1 ± 2.5
**14c**		88 ± 10			7.0 ± 1.9		2.6 ± 1.3
**15c**		89 ± 1			6.3 ± 1.8		4.5 ± 2.0
**16c**		82 ± 12			1.3 ± 0.4 *K_i_* = 1.9 ± 0.2 μM	53 ± 2	

Other test compounds including 13α-estrone (**9**), its 17-deoxy counterpart (**13**), and their halogenated derivatives (**10**–**12**, **14**–**16**) exerted very weak inhibitory effect: their relative conversion data are higher than 80% at a 10 µM test concentration. The empirical rules previously established in the 13β-series have not been observable in the 13α-estrone series, while the affinity for aromatase enzyme of the two basic 13α-estrone derivatives (**9** and **13**) could not be improved by attaching halogens onto ring A. This might be explained by the lack of ability of 13α-estrones for binding to the active site, because of their core-modified structure.

### STS

Numerous STS inhibitors have already been described in the literature^7^. Estrone aryl sulfamates are known as irreversible, suicide inhibitors. EMATE is a highly potent STS inhibitor, but because of its estrogenic activity it is not an adequate antitumor drug candidate. As literature data show the 17-deoxy analog of EMATE (NOMATE) displays similar STS inhibitory potential as its 17-keto counterpart^26^ ^,^ ^27^. This suggests that the presence of the 17-keto function is not essential for the effective inhibition of 3-sulfamates. E1 displays weak binding to STS, but its certain counterparts substituted in ring A exert substantial inhibition. This proves that appropriately substituted 3-OH E1 derivatives may also be good inhibitor candidates. It was established that substitution at C-4 of E1 with relatively small electron withdrawing-groups, such as F, Br, CN, formyl, or NO_2_, lead to improvement in inhibitory potency, which may be attributed to H-bonding and/or steric or other interactions. It is known that 4-formylestrone is a time- and concentration-dependent irreversible inhibitor of STS^7^, and it inactivates the enzyme by reacting with active site residues. Phan et al. proposed that the 4-formyl function is involved in Schiff base formation with amino groups in appropriate side-chains including Lys-368, Lys-134, and Arg-79^7^. As reported in the literature, the 3-OH function of the inhibitor may be involved in H-bonding with certain amino acid residues, with His346 and/or formylGly75 hydrate among others. Concerning hydrogen-bonding abilities, these side-chains are bifunctional. His346 may accept proton through its π-cloud and nitrogen, but may donate its NH proton. FormylGly75 hydrate may establish hydrogen bonds with its carbonyl group as a proton acceptor, whereas its OH group may behave as a proton donor. On the part of the steroid, both the OH function and its phenolate may form H-bonds or specific interactions. The electron-withdrawing properties of the introduced ring A substituents may greatly influence the polarisation and the acidity of the 3-OH group. This substituent effect depends on the position, number and nature of the introduced groups. Certain substituents at the *ortho* positions may additionally be involved in intramolecular H-bonding with the 3-OH group, which may reduce the affinity of the inhibitor to the enzyme. Phan et al. have not found direct correspondence between the estimated pK_a_ values (taken from the corresponding *o*-substituted phenols) and the inhibitor potentials of their examined compounds^7^.

Taking into account the above-mentioned literature results, it can be stated that not only the presence of a 3-*O*-sulfamoyl group but also the introduction of a relatively small electron-withdrawing group to carbon 4 of E1 may be a general possibility to enhance the potency of estrone-based STS inhibitors.

Here we start with the evaluation of the 2- and/or 4-chlorinated, brominated or iodinated 13β-estrone derivatives (**6a**,**b**,**c**–**8a**,**b**,**c**). The 4-iodo compound (**7a**) exerted outstanding submicromolar inhibition (Table 3). Its 0.23 µM IC_50_ value indicates a 22-fold higher affinity compared to the E1S substrate and an affinity increased by 100-fold compared to E1. **6a** its 2-sbstituted counterpart exerted 100-fold weaker inhibition according to its IC_50_ value. 4-Bromo derivative **7b** displayed submicromolar inhibitory potential with an IC_50_ value nearly twice as high as that of its 2-counterpart (**6b**). Phan et al. recently reported that the inhibition potential of **6c** is modest, whereas its 4-counterpart **7b** displays considerable inhibition^7^. Even so we found that both isomers are potent inhibitors. This difference may be ascribed to different substrates used in the two methods. Phan et al. used an artificial substrate (4-methylumbelliferyl sulfate), whereas we applied the natural substrate estrone-3-sulfate (**3**). The different binding specificity of these substrates may result in different inhibition results. Nevertheless, our results are in good agreement with those obtained recently by Phan et al. concerning the effect of the regioisomerism on the inhibitory potential^7^. The two chlorinated regioisomers (**6c** and **7c**) displayed commensurate inhibitory properties in the low micromolar range. Concerning all data obtained for the monosubstituted compounds (**6**, **7**), it can be stated that introduction of a large halogen substituent onto C-4 is particularly advantageous. The 2,4-*bis*-iodo compound (**8a**) had no marked influence on the conversion of E1S–E1. The *bis*-bromo compound (**8b**) displayed low micromolar inhibition, commensurable with that of its *bis*-chloro counterpart (**8c**).

With all compounds and their inhibitory data in hand we were additionally interested in the investigation of the potential correlation between their predicted pK_a_ values and the measured inhibitory potentials. pKa values were calculated using computer software available online^28^. Data are not listed separately. Predicted pK_a_ values suggest that our monosubstituted derivatives bear protonated 3-OH function (pK_a_ > 7.6), whereas *bis* compounds (pK_a_ = 6.8–7.4) are partially or completely deprotonated under the physiological conditions (pH =7.3) applied in our experiments. The obtained inhibition potentials do not have apparent direct relationship with the number and electronegativity of the introduced halogens, neither reflect the predicted pK_a_ data. Differences observed between inhibitory potentials of the two regioisomers indicate that electronic properties of the introduced halogens are not the determining factors in binding interactions of the 3-OH group^7^.

In the 13α-estrone series, the inhibitory potential of the compounds tested greatly depended on both the nature and the position of the halogen introduced. Introduction of iodine onto ring A was advantageous over substitution with smaller halogens (Br or Cl). 4-Bromo and 4-iodo compounds (**11a** and **11b**) were effective inhibitors with IC_50_ values of 8.5 and 6.0 µM. These regioisomers displayed better inhibitory potentials than their two counterparts (**10a** and **10b**). In the iodinated series, the best inhibitor was *bis*-iodo derivative **12a** with its low micromolar IC_50_ value. The chlorinated compounds (**10c**–**12c**) displayed weak inhibition with relative conversions over 50%.

The data obtained for the halogenated 17-deoxy-13α-estrone derivatives (**14**–**16**) reveal that all monohalogenated compounds (**14**, **15**), independently from the regioisomerism, are potent STS inhibitors in the low micromolar range. In general, these compounds displayed higher inhibitory potential than those of their 17-keto counterparts (**10**, **11**). We found that the presence of the 17-keto function on the halogenated 13α-estrane core is not essential for STS inhibitory activity. Additionally, empirical rules established previously do not predominate in this series. Furthermore, the IC_50_ values of the two regioisomers are comparable and the nature of introduced halogen is not crucial. Concerning the *bis*-substituted compounds (**16a**–**16c**), their inhibitory potential greatly depended on the nature of the halogen introduced. Surprisingly, the most potent **16c** compound in the 17-deoxy series, displaying an IC_50_ value of 1.3 µM, bears the smallest halogens. Compound **16c** exerts inhibition comparable to that of 4-bromoestrone **7b** (IC_50_ = 1.3 and 0.89 µM, respectively), but lower to that of 4-iodoestrone **7a** (IC_50_ = 0.23 µM).

**Figure 2.**
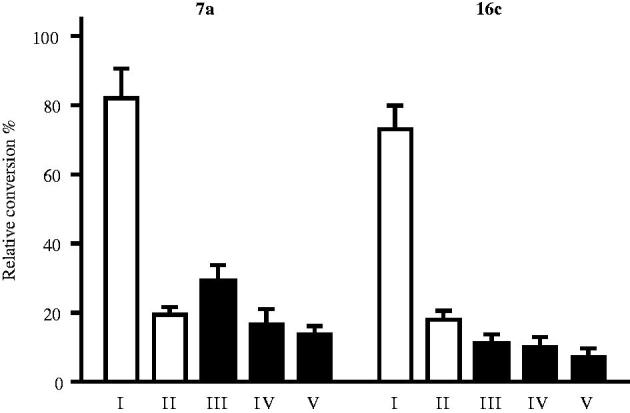
Investigation of STS inhibition reversibility of selected 13β-estrone compounds **7a**, **16c**. Inhibitor compounds were preincubated with human placental microsomes for 10 and 20 min. Following a 50-fold dilution step and 20 min secondary incubation to allow dissociation, the usual enzyme activity measurement was applied. Mean ± SD of three separate experiments. Experimental conditions: I No preincubation, **7a** 0.03 μM; **16c** 0.2 μM, II No preincubation, **7a** 1.5 μM; **16c** 10 μM, III Preincubation, 2.5 min, **7a** 1.5 μM; **16c** 10 μM, IV Preincubation, 10 min, **7a** 1.5 μM; **16c** 10 μM, V Preincubation, 20 min, **7a** 1.5 μM; 16c 10 μM.

The most potent representative test compounds **7a** and **16c** were selected for mechanistic and kinetic investigations. In the reversibility tests, placental microsomas were preincubated with high concentration of the inhibitor and enzyme activities were measured after dilution of the samples. Relative conversions in these preincubated samples (Figure 2, III and V) show suppressed enzyme activities, similar to those obtained for incubations with high inhibitor concentrations (Figure 2, II). Results indicate that **7a** and **16c** molecules do not dissociate upon dilution and they are bound to the enzyme in an irreversible manner. The time dependence of the reversibility test reveals that irreversible binding is nearly completed within a short 2.5 min time for both compounds (Figure 2, III).

**Figure 3.**
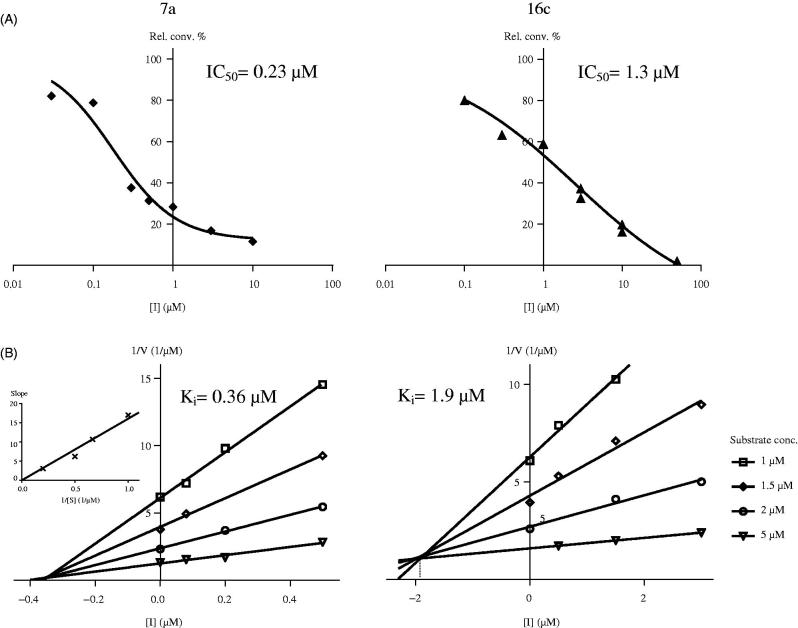
Concentration-dependent STS inhibition (A) and Dixon’s kinetic analysis (B) of selected 13β-estrone compounds **7a**, **16c**. Inset in B/7a shows the secondary plot of slopes of the Dixon’s lines vs. 1/substrate concentration.

Kinetic analyses of selected compounds **7a** and **16c** were performed by measurement of enzyme activities using different fixed substrate concentrations and varied inhibitor concentrations. Lines of the Dixon’s plots intersect in the second quadrant revealing competitive inhibition mechanisms (Figure 3, B). To confirm the competitive nature of the inhibition of **7a**, replot of slopes vs. 1/substrate concentration was constructed (inset B/**7a** in Figure 3). The resulting straight line passing through the origin supports the kinetics obtained from the Dixon’s plot^29^ ^,^ ^30^. Phan and coworkers investigated 4-nitroestrone as a representative estrone derivatives substituted at position C-4 with various electron-withdrawing functions. They have found the binding to be noncompetitive indicating that 4-nitroestrone is bound to the enzyme outside of its active site^7^. Our test compounds, 4-iodoestrone **7a** and 2,4-*bis*-chloro-17-deoxy-13α-estrone **16c** selected for the mechanistic investigations displayed a competitive mechanism, which alludes to binding within the active site of the enzyme.

K_i_ parameters were determined from the intersections of the Dixon’s plots, and these values were found to be 0.36 µM for **7a** and 1.9 µM for the **16c**. These measured *K_i_* values reflected inhibitory potentials ranked according to the IC_50_ data.

### 17β-hsd1

The crystal structure of 17β-HSD1 in its complex with E2 has been recently reported^31^. On the basis of this structure, molecular dynamic simulations and ligand–protein docking studies showed that there is an unoccupied lipophilic tunnel to the exterior of the protein located near the C-2 atom of E2^6^. These results may serve as the basis for the development of potent inhibitors, while the binding affinity of the potential inhibitor might be enhanced by introducing a lipophilic moiety to position 2. Möller *et al*.^6^ have found that 2-bromo and 2-chloro derivatives of E1 exerted potent inhibition and displayed similar binding affinity to that of the parent compound. We previously extended their research to the 13α-estrone series by performing brominations or iodinations on its aromatic ring^17^. All synthesised 3-OH derivatives (**10a**,**b**–**12a**,**b**) displayed outstanding low or submicromolar 17β-HSD1 inhibitory activities, but their IC_50_ values depended on the number, the nature and the position of the A-ring substituents (Table 3). The iodo derivatives (**10a**–**12a**) proved to be more potent than their bromo counterparts (**10b**–**12b**). The finding, that the 2,4-*bis*-iodo compound (**12a**) displays a submicromolar IC_50_ of 0.38 µM, was a novel result^17^.

Concerning that the iodo derivatives in the 13α-estrone series displayed high inhibitory potential, the iodo counterparts in the 13β-estrone series (**6a**–**8a**) have also been tested. Both iodo regioisomers (**6a**, **7a**) exerted outstanding inhibition, but the 2-iodo compound (**6a**) proved to be more potent with an IC_50_ value of 0.064 µM (Table 3). 2-Bromo- and 2-chloro-13β-estrone (**6b** and **6c**) exerted somewhat weaker potentials as their iodo counterpart (**6a**), nevertheless, we observed their improved binding affinity compared to the parent compound E1.

Efficiency of the 4-substituted counterparts (**7a**, **7b**) was found to be slightly weaker. These results are in a good agreement with empirical rules established previously concerning the two substitution to be up against the four substitution. New inhibition results of the iodo compounds (**6a**–**7a**) are particularly remarkable, and the inhibition potential of the 2-iodo regioisomer is outstanding. Additionally, all chlorinated derivatives (**6c**–**8c**) displayed outstanding commensurate submicromolar inhibition.

The three newly synthesised chloro 13α-estrone compounds (**10c**–**12c**) proved to be potent inhibitors. The 2-regioisomer (**10c**) seemed to be the most prominent with its submicromolar IC_50_ value of 0.33 µM. This value is commensurate with that of compound **6c**. This result indicates a twofold better binding than that of E1, and an affinity increased by fourfold in comparison to parent compound 13α-estrone **9**. The 4-chloro and the 2,4-*bis*-chloro derivatives (**11c** and **12c**) exerted somewhat weaker inhibitions (IC_50_ values were found to be 2.6 and 2.2 µM, respectively).

Comparing recent results obtained for bromo and iodo compounds in both the 13α- and 13β-series with those of the chloro-13α derivatives, it can be stated that there is less difference in the inhibitory potential of the two regioisomers in the 13α-series than in the natural estrone series except for the chloro-13α compounds. The inhibitory potential of 13α-estrone (**9**) increased four-fold by adding chlorine, a relatively small electron-withdrawing group, to C-2.

We were interested in the comparison of the inhibitory results concerning the 17-keto and the 17-deoxy-13α derivatives. The basic deoxy compound **13** displayed a surprising but outstanding inhibitory potential comparable with that of reference E1, the natural substrate of the enzyme. We expected that this promising result might be improved by the introduction of halogens to the aromatic ring of **13**. Nevertheless, the 1.1 µM IC_50_ value of **13** could not be lowered significantly. All halogenated derivatives (**14**–**16**) displayed comparable or higher values than that obtained for compound **13**. In this series the 2-halogenated compounds (**14a**–**c**) were found to be the best inhibitors (IC_50_ values 2.6, 1.3, and 2.9 µM, respectively). The 4-chloro (**15c**) and the 2,4-*bis*-bromo derivative (**16b**) exerted modest inhibition (IC_50_=4–5 µM), whereas other 17-deoxy-13α derivatives displayed weaker inhibition: their relative conversions exceeded 50% indicating IC_50_ > 10 µM.

It was established that the advantage of the 2-substitution over the 4-halogenation shows up in the 17-deoxy series, too. However, none of the new 17-deoxy inhibitor candidates displayed submicromolar IC_50_ values.

**Figure 4.**
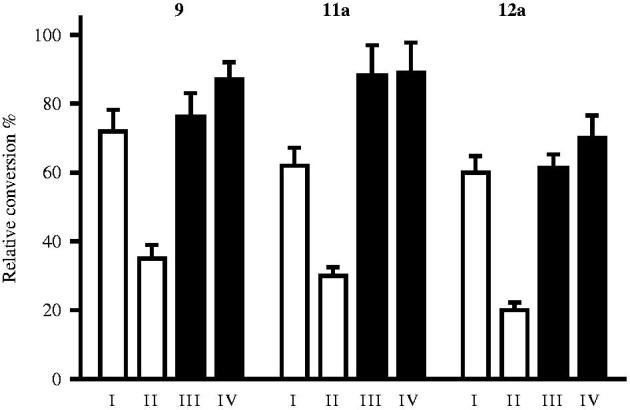
Investigation of 17β-HSD1 inhibition reversibility of selected 13α-estrone compounds **9**, **11a**, **12a**. Inhibitor compounds were preincubated with human placental cytosol. Following a 50-fold dilution step, the usual enzyme activity measurement was applied. Mean ± SD of three separate experiments. Experimental conditions: I No preincubation, 0.2 μM, II No preincubation, 10 μM, III Preincubation, 10 μM, 2.5 min, IV Preincubation, 10 μM, 20 min.

Potent 13α-estrone derivatives, basic compound **9**, its 4-iodo (**11a**) and 2,4-*bis*-iodo derivative **12a**, were selected for mechanistic and kinetic studies. To provide some information about the mechanism of action, we performed reversibility tests by preincubation of inhibitors with human placental cytosol. Figure 4 shows relative conversions obtained for the selected inhibitors according to preincubation conditions I–IV. The results indicate that the relative conversions in preincubated and diluted samples (Figure 4, III and IV), were similar to that obtained for incubation with the lower concentration of the inhibitor (Figure 4, I). This means that inhibitors **9**, **11a** and **12a** can be released from binding by dilution that is they bind to the enzyme in a reversible manner.

**Figure 5.**
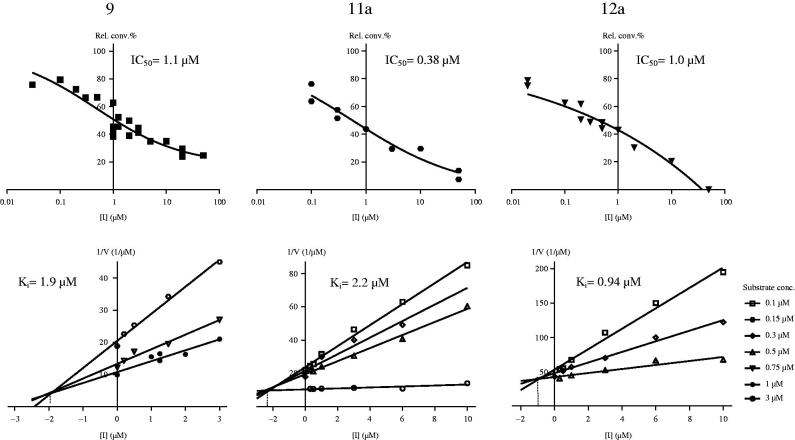
Concentration-dependent 17β-HSD1 inhibition (A) and Dixon’s kinetic analysis (B) of selected 13α-estrone compounds **9, 11a, 12a**.

In order to characterise the inhibition type and determine the K_i_, inhibition experiments were performed for the selected inhibitors **9**, **11a** and **12a** at different fixed substrate concentrations in the presence of cofactor excess. On the Dixon’s plot, data for each substrate concentration fall on straight lines which intersect in the second quadrant, alluding to competitive inhibition mechanism (Figure 5, B)^29^ ^,^ ^30^. Inhibitors that bind to the steroid site of 17β-HSD1 can bind to both the free enzyme and the binary enzyme–cofactor complex by random kinetic mechanism. This mixed-type inhibition, nevertheless, is simplified if the enzyme is saturated with cofactor first and displays competitive patterns^32^. Reversible and apparently competitive mechanism of the inhibition observed in our experiments shows that inhibitors **9**, **11a**, and **12a** are bound in the substrate-binding cavity of 17β-HSD1 with non-covalent interactions.

K_i_ parameters were determined from the intersections, and they were found to be 1.9, 2.2, and 0.94 µM for compounds **9**, **11a**, and **12a**, respectively. Inhibitory potentials on the basis of these *K_i_* data were comparable to those obtained by the IC_50_ values.

The 3-OH group of E1 and related inhibitors plays an important role in the binding to 17β-HSD1 forming a hydrogen-bonding system with the His221 and Glu282 residues at the recognition end of the active site^33^ ^,^ ^34^. However, literature data show high affinity of certain 3-OMe derivatives indicating that ligands may establish effective binding even in the absence of proton on the C-3 substituent^16^ ^,^ ^17^ ^,^ ^35–37^. Halogen substituents at C-2 and/or C-4 position modify binding abilities of the 3-OH substituent. The electron-withdrawing effect increases polarisation of the O–H bond and may induce deprotonation of this group under physiological pH conditions. *ortho* Substituents might also be able to form intramolecular hydrogen bond with the 3-OH group, which is disadvantageous^6^.

Our inhibition results demonstrate that introduction of halogen atoms to C-2 and/or to C-4 position increases affinity to 17β-HSD1. However, there does not appear to be a direct relationship between the number and electronegativity of the halogens and the inhibitor potency.

### Multiple or specific inhibition

Certain dual 17β-HSD1 and STS inhibitors were identified in both the 13β- and 13α-estrone series. Two 4-halo-17-keto-13β compounds (**7a** and **7b**) elicited submicromolar inhibitory effect towards both enzymes. Certain additional 17-keto compounds (**6b**, **6c**, **7c**, **8c**, **12a**) possess dual inhibitory properties with IC_50_ values in submicromolar or low micromolar range. In the 17-deoxy-13α-estrone series, all two-halogenated compounds (**14**), the 2,4-*bis*-bromo- (**16b**) and 4-chloro derivative (**15c**) exerted potent low micromolar dual action. Two compounds, namely 2-bromo- and 2-chloro-13β-estrones **6b** and **6c** exerted considerable inhibitions towards the three investigated enzymes.

It is interesting to note that inhibitory potentials of iodo derivatives in the 13β-estrone series display outstanding variations. 2-Iodo compound **6a** is a highly specific 17β-HSD1 inhibitor, but **7a** its 4-counterpart has dual STS and 17β-HSD1 inhibitory potential. The disubstituted derivative (**8a**) exerts weak effects towards the enzymes investigated.

Our results confirm that structurally different enzymes with distinct catalytic mechanisms might be inhibited by the same inhibitor compounds. Newly detected multiple 17β-HSD1, STS and/or aromatase inhibitors might be superior to compounds affecting the action of only a single enzyme. These multiple inhibitors may serve as good candidates for efficient suppression of local estrogen production in breast cancer tissues.

## Conclusions

Extensive research has been carried out in recent decades concerning enzyme inhibitors able to block estrogen biosynthesis. An armament of aromatase inhibitors is available by now in the medical practice; nevertheless, research work is continued to eliminate side effects and resistance developed by these medications^38^. Numerous compounds have also been evaluated as potential STS or 17β-HSD1 inhibitors, but these efforts have not been crowned with success, as none of the drug candidates has been clinically introduced for the treatment of estrogen-dependent diseases^3^ ^,^ ^32^ ^,^ ^39^. In order to develop potent new inhibitors of estrogen biosynthesis, a profound understanding of the enzymatic mechanisms and the structure–function relationships is essential. Our results obtained for aromatase, STS and 17β-HSD1 inhibition of 13α- and 13β-estrone compounds bearing halo substituents on their ring A make valuable contribution to this aim.

## Supplementary Material

Supplemental Material
