# Supplementary material for: Synthesis and structure–activity relationships of 2- and/or 4-halogenated 13β- and 13α-estrone derivatives as enzyme inhibitors of estrogen biosynthesis
Source: J Enzyme Inhib Med Chem. 2018 Sep 19;33(1):1271–82. doi: 10.1080/14756366.2018.1490731 (PMC6147116; doi:10.1080/14756366.2018.1490731)
Supplement: Supplemental Material [file IENZ_A_1490731_SM7826.pdf]

Supporting information for “Synthesis and structure–activity relationships of 2- and/or 4-halogenated 13β- and 13α-estrone derivatives as enzyme inhibitors of estrogen biosynthesis”

Ildikó Bacsa,[1,‡] Bianka Edina Herman,[2,‡] Rebeka Jójárt,[1] Kevin Stefán Herman,[1] János Wölfling,[1] Gyula Schneider,[1] Mónika Varga,[3] Csaba Tömböly,[4] Tea Lanišnik Rižner,[5] Mihály Szécsi,[2]* Erzsébet Mernyák[1]*

[‡] These authors contributed equally to this work.

[1] Department of Organic Chemistry, University of Szeged, Dóm tér 8, H-6720 Szeged, Hungary

[2] 1st Department of Medicine, University of Szeged, Korányi fasor 8–10, H-6720 Szeged, Hungary

[3] Department of Microbiology, University of Szeged, University of Szeged, H-6726 Szeged, Közép fasor 52, Hungary

[4] Laboratory of Chemical Biology, Institute of Biochemistry, Biological Research Centre of the Hungarian Academy of Sciences, P.O. Box 521, H-6701 Szeged, Hungary

[5] Institute of Biochemistry, Faculty of Medicine, University of Ljubljana, 1000 Ljubljana, Slovenia

*Corresponding authors. Tel.: +36 62 545825; fax: +36 62 545185 (M. Szécsi), tel.: +36 62 544277; fax: +36 62 544200 (E. Mernyák).

E-mail addresses: bobe@chem.u-szeged.hu (E. Mernyák), szecsi.mihaly@med.u-szeged.hu (M. Szécsi).

Content of this document

Experimental S2
- Chemical syntheses S2
  - General S2
  - Experimental conditions and characterization data S3
- Enzymatic assays S9
  - General S9
  - Preparation of enzyme sources S9
  - Incubation procedures S10
  - Inhibition studies S10
  - Reversibility studies S11

References S12

Experimental

Chemical syntheses

General

Melting points (mp) were determined with a Kofler hot-stage apparatus and are uncorrected. Elemental analyses were performed with a Perkin-Elmer CHN analyzer model 2400. Thin-layer chromatography: silica gel 60 F_{254}; layer thickness 0.2 mm (Merck); eluent: 30% EtOAc/hexane[a] or 30% diisopropylether/hexane[b]; detection with I_2 or UV (365 nm) after spraying with 5% phosphomolybdic acid in 50% aqueous phosphoric acid and heating at 100–120 °C for 10 min. Flash chromatography: silica gel 60, 40–63 μm (Merck). Reactions under microwave irradiation were carried out with a CEM Corporation focused microwave system, Model Discover SP. ^{1}H NMR spectra were recorded in DMSO-d_6, $CDCl_3$ solution with a Bruker DRX-500 instrument at 500 MHz, with Me_4Si as internal standard. ^{13}C NMR spectra were recorded with the same instrument at 125 MHz under the same conditions. Mass spectrometry, Method I.: Full scan mass spectra of the compounds were acquired in the range of 50 to 800 *m/z* with a Varian 500MS Ion trap mass spectrometer equipped with an electrospray ionization source. Analyses were performed in negative ion mode using flow injection mass spectrometry with a mobile phase of 50% aqueous acetonitrile containing 5 mM ammonium formate. The flow rate was 0.2 ml/min. Ten µl aliquot of the samples were loaded into the flow. The mass spectrometer was operated at a capillary and needle voltages of 80 and 5000 V, respectively. The RF loading was set at 88%. The nebulizing gas (N_2) and drying gas (N_2) pressures were maintained at 60 and 20 psi, respectively. Drying gas temperature was held at 300°C. Method II.: Full scan mass spectra of the compounds were acquired in the range 50 to 800 m/z with an Agilent 500MS Ion trap mass spectrometer equipped with an electrospray ionization source. Analyses were performed in positive ion mode. The spectra were collected by continous infusion of the steroid solution at a concentration of 10 ng ul-1 in MeCN/5 mM ammonium formate 50/50 (v/v %) at a flow rate of 15 ul min-1. The analytical HPLC measurements were performed on an Agilent 1260 Infinity HPLC equipped with a Micro Vacuum Degasser, Binary Pump, Standard Autosampler, Thermostatted Column Compartment, and Variable Wavelength Detector. The chromatographic separation was achieved on Gemini NX C-18 analytical column (3um, 150 x 2mm) from Phenomenex, equipped with a C-18 guard column, at 40 °C using gradient elution. Mobile phase A was water (Sigma Aldrich Ltd. Budapest, Hungary), while mobile

phase B was acetonitrile (Merck Ltd. Budapest, Hungary). A linear gradient was applied from 20% B to 100% B in 10 min (holding time: 5 min) then B content was lowered to 20% in 5 min and finally the column was re-equilibrated for 5 min. The flow rate was set to 0.2 ml/min.

Experimental conditions and characterization data

*3-Hydroxy-2-iodo-estra-1,3,5(10)-trien-17-one (**6a**), 3-hydroxy-4-iodo-estra-1,3,5(10)-trien-17-one (**7a**) and 3-hydroxy-2,4-diiodo-estra-1,3,5(10)-trien-17-one (**8a**)*

Compound **4** (200 mg, 0.74 mmol) was dissolved in trifluoroacetic acid (10 ml) and NIS (166 mg, 0.74 mmol) was added. The mixture was stirred at room temperature for 7 h, then poured onto 100 ml water and extracted with dichloromethane. The organic phase was separated, neutralized with ammonia solution and washed with a saturated solution of sodium thiosulfate and water. The organic phase was dried over anhydrous sodium sulfate, filtered and evaporated. The crude product was purified by flash chromatography with EtOAc/hexane = 15/85 as eluent. The first-eluted **7a** (49 mg, 17%) was obtained as a white solid. Mp.: 183–185°C [1]: R_f: 0.46[a]. Anal. Calcd. for $C_{18}H_{21}IO_2$: C, 54.56; H, 5.34; Found: C, 54.38; H, 5.45; ^{1}H NMR (500 MHz, DMSO-d_6) δ ppm: 0.81(s, 3H, 18-H_3); 2.58 and 2.79(2xm, 2x1H, 6-H_2); 6.69(d, 1H, J = 8.3 Hz, 2-H), 7.13(d, 1H, J = 8.3 Hz, 1-H); 10.01(s, 1H, OH); Continued elution yielded first a mixture of **7a** (38 mg, 13%) and **6a** (73 mg, 25%), and then compound **6a** (97 mg, 33%) as a white solid. Mp.: 205–207°C, [2]: 185°C, R_f: 0.37[a]. Anal. Calcd. for $C_{18}H_{21}IO_2$: C, 54.56; H, 5.34; Found: C, 54.43; H, 5.28; ^{1}H NMR (500 MHz, DMSO-d_6) δ ppm: 0.81(s, 3H, 18-H_3); 2.71(m, 2H, 6-H_2); 6.58(s, 1H, 4-H); 7.47(s, 1H, 1-H); 9.91(s, 1H, OH);

Compound **4** (100 mg, 0.37 mmol) was dissolved in trifluoroacetic acid (5 ml), and NIS (166 mg, 0.74 mmol) was added. The mixture was stirred at room temperature for 3.5 h, and then poured onto 100 ml water and extracted with dichloromethane. The organic phase was separated, neutralized with ammonia solution and washed with a saturated solution of sodium thiosulfate and water. The organic phase was dried over anhydrous sodium sulfate, filtered and evaporated. The crude product was purified by flash chromatography with EtOAc/hexane = 10/90 as eluent. The first-eluted **8a** (57 mg, 30%) was obtained as a white solid. Mp.: 200–202 °C [3], R_f: 0.48[a]. Anal. Calcd. for $C_{18}H_{21}I_2O_2$: C, 41.40; H, 3.86; Found: C, 41.58; H, 3.95; ^{1}H NMR (500 MHz, $CDCl_3$) δ ppm: 0.90(s, 3H, 18-H_3); 2.68 and 2.85(2xm, 2x1H 6-

H_2); 5.78(s, 1H, OH); 7.62(s, 1H, 1-H). Continued elution yielded compound **7a** (87 mg, 59%) as a white solid.

*2-Bromo-3-hydroxy-estra-1,3,5(10)-trien-17-one (**6b**), 4-bromo-3-hydroxy-estra-1,3,5(10)-trien-17-one (**7b**) and 2,4-dibromo-3-hydroxy-estra-1,3,5(10)-trien-17-one (**8b**)*

Compound **4** (200 mg, 0.74 mmol) was dissolved in CH_2Cl_2 (6 ml), and NBS (132 mg, 0.74 mmol) was added. The mixture was stirred at rt for 2 h. The solvent was then evaporated off, and the crude product was purified by flash chromatography with EtOAc/hexane = 0.5/95 as eluent. The first-eluted **7b** (60 mg, 23%) was obtained as a white solid. Mp.: 220–222°C, [4]: 264–265°C, from ethanol, R_f: 0.47[a]. Anal. Calcd. for $C_{18}H_{21}BrO_2$: C, 61.90; H, 6.06. Found: C, 62.05; H, 6.18; ^1H NMR (500 MHz, DMSO-d_6) δ ppm: 0.81(s, 3H, 18-H_3); 2.61 and 2.85(2xm, 2x1H, 6-H_2); 6.75(d, 1H, J = 8.4 Hz, 2-H), 7.12(d, 1H, J = 8.4 Hz, 1-H); 9.90(s, 1H, OH); (Continued elution yielded first a mixture of **7b** (46 mg, 18%) and **6b** (65 mg, 25%), and then compound **6b** (39 mg, 15%) as a white solid. Mp.: 206–208 °C, [5]: 262–264°C, R_f: 0.44[a] Anal. Calcd. for $C_{18}H_{21}BrO_2$: C, 61.90; H, 6.06; Found: C, 61.87; H, 5.92; ^1H NMR (500 MHz, DMSO-d_6) δ ppm: 0.82(s, 3H, 18-H_3); 2.71(m, 2H, 6-H_2); 6.64(s, 1H, 4-H); 7.28(s, 1H, 1-H); 9.82(s, 1H, OH);

Compound **4** (100 mg, 0.37 mmol) was dissolved in CH_2Cl_2 (5 ml), and NBS (132 mg, 0.74 mmol) was added. The mixture was stirred at rt for 6 h. The solvent was then evaporated off, and the crude product was purified by flash chromatography with EtOAc/hexane = 10/90 as eluent. The first-eluted **8b** (63 mg, 39%) was obtained as a white solid. Mp.: 219–221°C [6]: 224–226°C, R_f: 0.53[a]. Anal. Calcd. for $C_{18}H_{21}Br_2O_2$: C, 50.49; H, 4.71; Found: C, 50.62; H, 4.83; ^1H NMR (500 MHz, $CDCl_3$) δ ppm: 0.90(s, 3H, 18-H_3); 2.68 and 2.94(2xm, 2x1H 6-H_2); 5.84(s, 1H, OH); 7.41(s, 1H, 1-H). Continued elution yielded first a mixture of **8b** (53 mg, 33%) and **7b** (10 mg, 8%), and then compound **7b** (5 mg, 4%) as a white solid.

*2-Chloro-3-hydroxy-estra-1,3,5(10)-trien-17-one (**6c**), 4-chloro-3-hydroxy-estra-1,3,5(10)-trien-17-one (**7c**) and 2,4-dichloro-3-hydroxy-estra-1,3,5(10)-trien-17-one (**8c**)*

Estrone **4** (100 mg, 0.37 mmol) was dissolved in the mixture of acetonitrile (4 ml), then trifluoroacetic acid (0.004 ml) and *N*-chlorosuccinimide (NCS; 54.5 mg, 0.41 mmol) were

added. The mixture was stirred at 80 °C for 1 h. Then the solvent was evaporated off and the crude product was purified by flash chromatography with EtOAc/hexane = 10/90 as eluent. The first-eluted **7c** (28 mg, 25%) was obtained as a white solid. Mp.: 180–182°C [6]: 272–274°C, R_f: 0.46[a]. Anal. Calcd. for $C_{18}H_{21}ClO_2$: C, 70.93; H, 6.94; Found: C, 78.08; H, 7.05; ^{1}H NMR (500 MHz, $CDCl_3$) δ ppm: 0.90(s, 3H, 18-H_3); 2.74 and 3.01(2xm, 2x1H, 6-H_2); 5.51(s, 1H, OH); 6.86(d, 1H, *J* = 8.6 Hz, 2-H), 7.18(d, 1H, *J* = 8.6 Hz, 1-H). Continued elution yielded the mixture of compounds **7c** (23 mg, 20%) and **6c** (18 mg, 16%). Finally eluted **6c** (16 mg, 14%) obtained as a white solid. Mp.: 203–205 °C, [5]: 262–264°C, R_f: 0.42[a]; Anal. Calcd. for $C_{18}H_{21}ClO_2$: C, 70.93; H, 6.94; Found: C, 71.12; H, 6.84; ^{1}H NMR (500 MHz, $CDCl_3$) δ ppm: 0.91(s, 3H, 18-H_3); 2.85(m, 2H, 6-H_2); 5.32(s, 1H, OH); 6.75(s, 1H, 4-H); 7.21(s, 1H, 1-H).

Estrone **4** (100 mg, 0.37 mmol) was dissolved in dichloromethane (5 ml) and *N*-chlorosuccinimide (NCS; 109 mg, 0.81 mmol) was added. The mixture was stirred at rt for 6 h. The solvent was then evaporated off, and the crude product was purified by flash chromatography with EtOAc/hexane = 10/90 as eluent. The first-eluted **8c** (34 mg, 27%) was obtained as a white solid. Mp.: 195–197 °C, [6]: 212–213°C, R_f: 0.48[a]. Anal. Calcd. for $C_{18}H_{21}Cl_2O_2$: C, 63.79; H, 5.94; Found: C, 63.86; H, 6.12;^{1}H NMR (500 MHz, $CDCl_3$) δ ppm: 0.91(s, 3H, 18-H_3); 2.72 and 2.98(2xm, 2x1H 6-H_2); 5.75(s, 1H, OH); 7.22(s, 1H, 1-H). Continued elution yielded the mixture of compounds **8c** (17 mg, 13%) and **7c** (18 mg, 16%). Finally eluted **7c** (28 mg, 24%) obtained as a white solid.

*2-Chloro-3-hydroxy-13α-estra-1,3,5(10)-trien-17-one (**10c**), 4-chloro-3-hydroxy-13α-estra-1,3,5(10)-trien-17-one (**11c**) and 2,4-dichloro-13α-3-hydroxy-estra-1,3,5(10)-trien-17-one (**12c**)*

13α-Estrone **9** (135 mg, 0.50 mmol) was dissolved in trifluoroacetic acid (5 ml) and *N*-chlorosuccinimide (NCS; 67 mg, 0.50 mmol) was added. at 0 °C. After warming up to room temperature the mixture was stirred for 24 h and then poured onto 100 ml water and extracted with diethyl ether. The organic phase was dried over anhydrous sodium sulfate, filtered and evaporated. The crude product was purified by flash chromatography with diisopropyl ether/hexane = 30/70 as eluent. The first-eluted **11c** (95 mg, 63%) was obtained as a white solid. Mp.: 209–211 °C, R_f: 0.20[b]. Anal. Calcd. for: $C_{18}H_{21}ClO_2$: C, 70.93; H, 6.94; Found: C,

78.02; H, 6.85; ^{1}H NMR (500 MHz, DMSO-d_6) δ ppm: 0.96(s, 3H, 18-H_3); 2.58 and 2.85(2xm, 2H, 6-H_2); 6.75(d, 1H, J = 8.5 Hz 4-H); 7.06(d, 1H, J = 8.5 Hz , 1-H); 9.73(s, 1H, OH); ^{13}C NMR (125 MHz, $CDCl_3$) δ ppm 21.0; 25.0(C-18); 27.9; 28.0; 28.4; 31.9; 33.4; 40.7; 41.6; 49.0; 50.0(C-13); 113.1(C-2); 119.9(C-4); 125.3(C-1); 133.3(C-10); 134.9(C-5); 149.2(C-3); 221.5(C-17). MS^I m/z (%): 303 (100, $[M – H]^–$). Continued elution yielded compound **10c** (24 mg, 16%) obtained as a white solid. Mp.: 224–226 °C, R_f: 0.13[b] Anal. Calcd. for $C_{18}H_{21}ClO_2$: C, 70.93; H, 6.94; Found: C, 70.86; H, 6.75; ^{1}H NMR (500 MHz, DMSO-d_6) δ ppm: 0.96(s, 3H, 18-H_3); 2.67(m, 2H, 6-H_2); 6.62(s, 1H, 4-H); 7.14(s, 1H, 1-H); 9.75(s, 1H, OH); ^{13}C NMR (125 MHz, DMSO-d_6) δ ppm: 20.4; 24.4(C-18); 27.5; 27.9; 29.0; 31.5; 32.8; 40.4; 40.5; 49.3(C-13); 116.1(C-4); 116.9(C-2); 126.8(C-1); 131.5(C-10); 136.5(C-5); 150.4(C-3); 220.5(C-17) MS^I m/z (%): 303 (100, $[M – H]^–$).

13α-Estrone **9** (135 mg, 0.50 mmol) was dissolved in trifluoroacetic acid (5 ml) and *N*-chlorosuccinimide (NCS; 134 mg, 1.00 mmol) was added at 0 °C. After warming up to room temperature the mixture was stirred for 24 h and then poured onto 100 ml water and extracted with diethyl ether. The organic phase was dried over anhydrous sodium sulfate, filtered and evaporated. The crude product was purified by flash chromatography with diisopropyl ether/hexane = 30/70 as eluent. The first-eluted **12c** (19 mg, 11%) was obtained as a white solid. Mp.: 166–168 °C, R_f: 0.24[b]; Anal. Calcd. for $C_{18}H_{20}Cl_2O_2$: C, 63.73; H, 5.94; Found: C, 63.62; H, 6.04; ^{1}H NMR (500 MHz, DMSO-d_6) δ ppm: 1.06(s, 3H, 18-H_3); 2.63 and 2.94(2xm, 2H, 6-H_2); 5.74(s, 1H, OH); 7.19(s, 1H, 1-H); ^{13}C NMR (125 MHz, $CDCl_3$) δ ppm 21.0; 25.0(C-18); 27.8; 28.2; 28.3; 31.8; 33.3; 40.5; 41.5; 48.9; 49.9(C-13); 118.0 and 120.8(C-2 and C-4); 125.3(C-1); 133.8(C-10); 134.4(C-5); 145.5(C-3); 221.2(C-17); MS^I m/z (%): 337 (100, $[M – H]^–$). Continued elution yielded a mixture of compound **12c** (13 mg, 8%) and compound **11c** (24 mg, 16%). Finally eluted compound **11c** (62 mg, 41%) obtained as a white solid.

3-Hydroxy-2-iodo-13α-estra-1,3,5(10)-triene ***(14a)****, 3-hydroxy-4-iodo-13α-estra-1,3,5(10)-triene* ***(15a)****, 3-hydroxy-2,4-diiodo-13α-estra-1,3,5(10)-triene* ***(16a)***

17-Deoxy-13α-estrone **13** (128 mg, 0.50 mmol) was dissolved in trifluoroacetic acid (5 ml) and *N*-iodosuccinimide (NIS; 169 mg, 0.75 mmol) was added. The mixture was stirred at room temperature for 2 h, then poured onto 100 ml water and extracted with dichloromethane. The organic phase was separated, neutralized with ammonia solution and washed with a saturated solution of sodium thiosulfate and water. The organic phase was dried over

anhydrous sodium sulfate, filtered and evaporated. The crude product was subjected to flash chromatography. The first-eluted **16a** (61 mg, 24%) was obtained as a white solid. Mp.: 145–148 °C, R_f: 0.79[b]; Anal. Calcd. for $C_{18}H_{22}I_2O$: C, 42.54; H, 4.36; Found: C, 42.38; H, 4.45; ^{1}H NMR (500 MHz, DMSO-d_6) δ ppm: 0.91(s, 3H, 18-H_3); 2.46 és 2.69 (2xm, 2x1H, 6-H_2); 7.63(s, 1H, 1-H); 9.21(s, 1H, OH); ^{13}C NMR (125 MHz, $CDCl_3$) δ ppm:20.9; 27.2; 27.8; 28.9; 29.8(C-18); 33.4; 35.5; 37.8; 40.2; 41.7(C-13); 42.3; 51.4; 78.3(C-2); 91.8(C-4); 136.4(C-1); 137.0(C-10); 141.3(C-5); 151.0(C-3). MS[I] m/z (%): 381 (100, $[M – H]^-$). Continued elution yielded **15a** (28 mg, 15%) obtained as a white solid. Mp.: 91–92 °C, R_f: 0.58[b]. Anal. Calcd. for $C_{18}H_{23}IO$: C, 56.55; H, 6.06; Found: C, 56.72; H, 5.91; ^{1}H NMR (500 MHz, $CDCl_3$) δ ppm: 0.96(s, 3H, 18-H_3); 2.62 és 2.82 (2xm, 2x1H, 6- H_2); 5.35(s, 1H, OH); 6.84(d, 1H, J = 8.3 Hz, 2-H); 7.23(d, 1H, J = 8.3 Hz, 1-H); ^{13}C NMR (125 MHz, $CDCl_3$) δ ppm: 20.9; 27.3; 27.8; 29.1; 29.9(C-18); 33.5; 35.7; 37.8; 40.6; 41.8(C-13); 42.6; 51.5; 95.0(C-4); 111.9(C-2); 127.4(C-1); 134.9(C-10); 139.9(C-5); 152.5(C-3). MS[I] m/z (%): 381 (100, $[M – H]^-$). Continued elution yielded first a mixture of **15a** (18 mg, 9%) and **14a** (31 mg, 16%), and finally compound **14a** (61 mg, 32%) as a white solid. Mp.: 60–63°C, R_f: 0.47[b]. Anal. Calcd. for $C_{18}H_{23}IO$: C, 56.55; H, 6.06; Found: C, 56.68; H, 6.12; ^{1}H NMR (500 MHz, $CDCl_3$) δ ppm: 0.96(s, 3H, 18-H_3); 2.74(m, 2H, 6-H_2); 5.08(s, 1H, OH); 6.70(s, 1H, 4-H); 7.55(s, 1H, 1-H); ^{13}C NMR (125 MHz, $CDCl_3$) δ ppm: 20.9; 27.0; 27.8; 28.1; 29.9(C-18); 30.1; 33.5; 35.6; 40.9; 41.8(C-13); 42.0; 51.6; 82.5(C-2); 114.7(C-4); 135.6(C-1); 135.7(C-10); 139.8(C-5); 152.3(C-3). MS[I] m/z (%): 507 (100, $[M – H]^-$).

*2-Bromo-3-hydroxy-13α-estra-1,3,5(10)-triene (**14b**), 4-bromo-3-hydroxy-13α-estra-1,3,5(10)-triene (**15b**), 2,4-dibromo-3-hydroxy-13α-estra-1,3,5(10)-triene (**16b**)*

17-Deoxy-13α-estrone **13** (128 mg, 0.50 mmol) was dissolved in dichloromethane (5 ml) and *N*-bromosuccinimide (NBS; 98 mg, 0.55 mmol) was added. The mixture was stirred at rt for 1 ~~2–3~~ h, the solvent was then evaporated off, and the crude product was purified by flash chromatography with EtOAc/hexane = 5/95 as eluent. The first-eluted **16b** (23 mg, 11%) was obtained as a white solid. Mp.: 120–123°C, R_f: 0.81[b]. Anal. Calcd. for $C_{18}H_{22}Br_2O$: C, 52.20; H, 5.35; Found: C, 52.35; H, 5.41; ^{1}H NMR (500 MHz, $CDCl_3$) δ ppm: 0.96(s, 3H, 18-H_3); 2.58 és 2.87 (2xm, 2x1H, 6-H_2); 5.81(s, 1H, OH); 7.44(s, 1H, 1-H); ^{13}C NMR (125 MHz, $CDCl_3$) δ ppm: 20.9; 27.2; 27.8; 28.2; 29.9(C-18); 31.8; 33.4; 35.6; 40.2; 41.8(C-13); 42.4; 51.5; 106.4(C-2); 112.9(C-4); 129.1(C-1); 136.1 and 137.2(C-5 and C-10); 146.9(C-3). MS[II] *m/z* (%): 390 (100); 413 (45, $[M + H]^+$). Continued elution yielded **15b** (32 mg, 19%) obtained as a white solid. Mp.: 111–114°C, R_f: 0.62[b]. Anal. Calcd. for $C_{18}H_{23}BrO$: C, 64.48;

H, 6.91; Found: C, 64.52; H, 7.04; ^{1}H NMR (500 MHz, DMSO-d_6) δ ppm: 0.93(s, 3H, 18-H_3); 2.52 és 2.80 (2xm, 2x1H, 6- H_2); 6.75(d, 1H, *J*=8.2 Hz, 2-H); 7.14(d, 1H, *J*=8.2 Hz, 1-H); 9.81(s, 1H, OH); ^{13}C NMR (125 MHz, DMSO-d_6) δ ppm: 20.4; 26.7; 27.2; 27.9; 29.6(C-18); 31.5; 32.9; 35.3; 39.9; 41.4(C-13); 42.0; 50.9; 112.1(C-4); 113.2(C-2); 125.5(C-1); 132.7(C-10); 136.7(C-5); 151.6(C-3). MS[I] *m/z* (%): 333 (100, $[M – H]^{–}$). Continued elution yielded first a mixture of **15b** (23 mg, 14%) and **14b** (42 mg, 25%), and finally compound **14b** (32 mg, 19%) obtained as a white solid. Mp.: 75–77°C, R_f: 0.56[b]. Anal. Calcd. for $C_{18}H_{23}BrO$: C, 64.48; H, 6.91; Found: C, 64.61; H, 7.08, ^{1}H NMR (500 MHz, DMSO-d_6) δ ppm: 0.96(s, 3H, 18-H_3); 2.63(m, 2H, 6-H_2); 6.61(s, 1H, 4-H); 7.30(s, 1H, 1-H); 9.78(s, 1H, OH); ^{13}C NMR (125 MHz, DMSO-d_6) δ ppm: 20.3; 26.4; 27.2; 27.6; 29.3; 29.6(C-18); 33.0; 35.2; 40.4; 41.3(C-13); 41.4; 51.0; 106.5(C-2); 115.8(C-4); 129.7(C-1); 132.6(C-10); 137.1(C-5); 151.3(C-3). MS[I] *m/z* (%):333 (100, $[M – H]^{–}$).

*2-Chloro-3-hydroxy-13α-estra-1,3,5(10)-triene (**14b**), 4-chloro-3-hydroxy-13α-estra-1,3,5(10)-triene (**15b**), 2,4-dichloro-3-hydroxy-13α-estra-1,3,5(10)-triene (**16b**)*

17-Deoxy-13α-estrone **13** (128 mg, 0.50 mmol) was dissolved in acetonitrile (5 ml) and *N*-chlorosuccinimide (NCS; 100 mg, 0.75 mmol) was added. The mixture was heated in a CEM microwave reactor at 80 °C for 40 min under stirring. The crude product was purified by flash chromatography with diisopropyl ether/hexane = 30/70 as eluent. The first-eluted **15c** (23 mg, 16%) was obtained as a white solid. Mp.: 125–128 °C, R_f: 0.60[b]. Anal. Calcd. for $C_{18}H_{23}ClO$: C, 74.34; H, 7.97; Found: C, 73.54; H, 8.15; ^{1}H NMR (500 MHz, DMSO-d_6) δ ppm: 0.92(s, 3H, 18-H_3); 2.52 és 2.82(2xm, 2x1H, 6-H_2); 6.76(d, 1H, *J* = 8.3 Hz, 2-H); 7.09(d, 1H, *J* = 8.3 Hz, 1-H); 9.69(s, 1H, OH); ^{13}C NMR (125 MHz, DMSO-d_6) δ ppm: 20.4; 26.6; 27.2; 27.6; 28.3; 29.6(C-18); 32.9; 35.2; 39.8; 41.3(C-13); 41.8; 50.9; 113.4(C-2); 120.4(C-4); 124.6(C-1); 132.3(C-10); 135.1(C-5); 150.6(C-3). MS[I] m/z (%): 289 (100, $[M – H]^{–}$). Continued elution yielded first a mixture of **15c** (12 mg, 8%) and **14c** (22 mg, 15%), and finally compound **14c** (48 mg, 33%) obtained as a white solid. Mp.: glassy, R_f: 0.52[b]. Anal. Calcd. for $C_{18}H_{23}ClO$: C, 74.34; H, 7.97; Found: C, 74.28; H, 8.13; ^{1}H NMR (500 MHz, DMSO-d_6) δ ppm: 0.92(s, 3H, 18-H_3); 2.65(m, 2H, 6-H_2); 6.62(s, 1H, 4-H); 7.17(s, 1H, 1-H); 9.71(s, 1H, OH); ^{13}C NMR (125 MHz, DMSO-d_6) δ ppm: 20.3; 26.4; 27.2; 27.7; 29.2; 29.7(C-18); 33.0; 35.2; 40.4; 41.4(C-13); 41.5; 51.0; 116.1(C-4); 116.9(C-2); 126.8(C-1); 132.1(C-10); 136.4(C-5); 150.3(C-3). MS[II] *m/z* (%): 289 (100, M^{+}).

17-Deoxy-13α-estrone **13** (128 mg, 0.50 mmol) was dissolved in acetonitrile (5 ml) and *N*-chlorosuccinimide (NCS; 200 mg, 1.5 mmol) was added. The mixture was heated in a CEM microwave reactor at 80 °C for 40 min under stirring The crude product was purified by flash chromatography with diisopropyl ether/hexane = 30/70 as eluent. The first-eluted **16c** (85 mg, 53%) was obtained as a white solid. Mp.: 100–103 °C, R_f: 0.84[b]. Anal. Calcd. for $C_{18}H_{22}Cl_2O$: C, 66.47; H, 6.82; Found: C, 66.62; H, 6.97; ^{1}H NMR (500 MHz, DMSO-d_6) δ ppm: 0.92(s, 3H, 18-H_3); 2.55 és 2.80(2xm, 2x1H, 6-H_2); 7.27(s, 1H, 1-H); 9.66(s, 1H, OH); ^{13}C NMR (125 MHz, DMSO-d_6) δ ppm: 20.7; 26.8; 27.5; 27.6; 28.6; 29.9(C-18); 33.2; 35.4; 39.9; 41.7(C-13); 42.0; 51.2; 119.4 and 122.5(C-2 and C-4); 125.6(C-1); 134.3(C-10); 134.8(C-5); 146.8(C-3) MS[I] m/z (%): 353 (100), 323 (34, $[M-H]^-$). Continued elution yielded compound **15c** (38 mg, 26%) obtained as a white solid.

Enzymatic assays

General

The ability of the synthesized compounds to inhibit activity of aromatase, steroid sulfatase (STS) and 17β-hydroxysteroid dehydrogenase type 1 (17β-HSD1) was examined. *In vitro* radiosubstrate incubations were performed with adapted literature methods and human placenta served as source of the enzymes. (References: for aromatase [7–9], for STS [10] and for 17β-HSD1 [11, 12]). Radiolabelled steroids were obtained from American Radiolabeled Chemicals, St. Louis, MO, USA). Other chemicals and solvents of analytical grade purity were purchased from Sigma (St. Louis, MO, USA), from Fluka (Buchs, Switzerland) or from Merck (Darmstadt, Germany).

Preparation of enzyme sources

Human term placentae were collected immediately after delivery and stored frozen at -80 °C. Tissue specimens were homogenized with an Ultra-Turrax in 0.1 M HEPES buffer solution (pH=7.3) containing 1 mM EDTA and 1 mM dithiotreitol, and cytosol and microsomas were obtained with fractionated centrifugation. Application of the human tissue was approved by the institutional Human Investigation Review Board.

Incubation procedures

Enzymatic incubations were carried out in the HEPES buffer medium at a final volume of 200 μl. The substrate was added to the incubate in 20 μl of a 25 v/v% propylene glycol in HEPES buffer solution, whereas test compounds were applied in 10 μl of dimethyl sulfoxide solution. Incubations were performed at 37 °C and terminated by cooling and the addition of organic solvents of the subsequent extraction procedure. Control samples with no inhibitor and blank samples were incubated simultaneously. The radioactivity of the products formed was measured by means of liquid scintillation counting (LSC) and taken as indicator of the enzyme activity.

In the aromatase assays the microsoma suspension was incubated with appropriate concentration of unlabelled testosterone (**2**) substrate spiked with [1,2,6,7-^{3}H]testosterone tracer, and in the presence of 0.1 mM NADPH cofactor excess. (In this case, solution of the substrate was prepared with acetonitrile instead of propylene glycol.) Incubation time was 40 min. The incubation mixture was extracted with toluene, then the toluene phase was drained and washed with HEPES buffer. Aromatase products containing phenolic hydroxy group were extracted with 1.2 M sodium hydroxide solution from the toluene extract and the alkaline phase was measured in the LSC.

STS assays were performed using microsoma suspension and estrone-3-sulfate (E1S, **3**) substrate with [6,7-^{3}H]estrone-3-sulfate tracer with incubations lasting for 20 min. Reactions were terminated by the addition of equal volume of methanol, and the product estrone (E1, **4**) was extracted with toluene.

In the 17β-HSD1 assays the cytosol were incubated using estrone (E1, **4**) substrate with [6,7-^{3}H]estrone tracer and 0.1 mM NADPH cofactor excess. The incubation time was 2.5 min. The product 17β-estradiol (E2, **5**) was extracted with ethyl acetate and isolated by a TLC method (details are published in our earlier reference [12]).

Inhibition studies

In the general procedure, incubations were performed as described. Concentrations of the substrates were 1.0 μM, and the test compounds were applied at 10 μM final concentration in the incubate. Relative conversions compared to non-inhibited controls (100%) were determined. The assays were performed in triplicate, and the mean value and the standard deviation (SD) were calculated.

IC_{50} values (the inhibitor concentration that decreases the enzyme activity to 50%) were determined for the most effective test compounds and for certain reference compounds. In these cases, conversions were measured at 10–15 different concentrations in the appropriate interval between 0.001–50 μM. IC_{50} results were calculated by using unweighted iterative least squares logistic curve fitting by means of the "absolute IC_{50} calculation" function of the GraphPad Prism 4.0 software (GraphPad Software, Inc., San Diego, CA, USA).
Kinetic analyses were performed via measurement of the enzymatic transformation using different fixed substrate concentrations and varied inhibitor concentrations. The Dixon's linear transformation (1/velocity *vs.* inhibitor concentration) was applied for evaluation and the inhibition constant (K_i) was determined from abscissa of intersection of the lines of different substrate concentrations. Mechanism of binding was identified according to the Dixon's graphs and the secondary plot of slopes of the Dixon's lines *vs.* 1/substrate concentration.

Reversibility studies

The inhibitor compounds were preincubated with the appropriate placenta fraction in a volume of 4.0 µl at 37 °C for various time periods (2.5-40 min). Following this procedure, samples were diluted with the incubation medium to 50-fold. Enzyme activity measurements were started immediately for the 17β-HSD1 and after 20 min secondary incubation time to allow dissociation in the case of STS. The enzymatic reactions were started by the addition of the cofactor and the substrate, and subsequent incubation procedures were then carried out as described. Conversions were compared to their respective controls, which were treated in a similar way but were made without inhibitors.

References

[1] M. Numazawa, Y. Ogura, K. Kimura, M. Nagaoka, Synthesis of 2-methoxy- and 4-methoxyestrogens with halogen-methoxy exchange reactions, J. Chem. Research (S). 11 (1985) 348–349.

[2] A. Hillmann-Elies, G. Hillmann, U. Schiedt, 2-Jod-östron und 2-jod-östradiol, Zeitschrift für Naturforschung, 8 b (1953): 436–440.

[3] M. Numazawa, Y. Ogura, Efficient synthesis of 2-methoxy- and 4-methoxy-estrogens, J. Chem. Soc., Chem. Commun., 9 (1983) 533–534.

[4] J. S. Mitchell, Y. Wu, C. J. Cook, L. Main, Estrogen conjugation and antibody binding interactions in surface plasmon resonance biosensing, Steroids 71 (2006) 618–631.

[5] H. Ali, J. E. Lier, Reaction of benzeneselenenyl halides with estrogens, J. Chem. Soc. Perkin Trans. 1 (1991) 269-271.

[6] E. Schwenk, C. G. Castle, G. E. Joachim, Halogenation of estrone and derivatives, J. Org. Chem. 28 (1963) 136–144.

[7] E. A. Thompson Jr., P. K. Siiteri, Utilization of oxygen and reduced nicotinamide adenine dinucleotide phosphate by human placental microsomes during aromatization of androstenedione, J. Biol. Chem. 249 (1974) 5364–5372.

[8] J. T. Kellis Jr., L. E. Vickery, Purification and characterization of human placental aromatase cytochrome P-450, J. Biol. Chem. 262 (1987) 4413–4420.

[9] A. LaMorte, N. Kumar, C. W. Bardin, K. Sundaram, Aromatization of 7 alpha-methyl-19-nortestosterone by human placental microsomes in vitro, J. Steroid Biochem. Mol. Biol. 48 (1994) 297–304.

[10] K. K. Leslie, D. J. Zuckerman, J. Schruefer, M. Burchell, J. Smith, B. D. Albertson, Oestrogen modulation with parturition in the human placenta. Placenta 15 (1994) 79–88.

[11] M. R. Tremblay, D. Poirier, Overview of a rational approach to design type I 17beta-hydroxysteroid dehydrogenase inhibitors without estrogenic activity: chemical synthesis and biological evaluation, J. Steroid Biochem. Mol. Biol. 66 (1998) 179–191.

[12] B. E. Herman, J. Szabó, I. Bacsa, J. Wölfling, G. Schneider, M. Bálint, C. Hetényi, E. Mernyák, M. Szécsi, Comparative investigation of the in vitro inhibitory potencies of 13-epimeric estrones and D-secoestrones towards 17β-hydroxysteroid dehydrogenase type 1, J. Enzyme Inhib. Med. Chem. 31 (2016) 61–69. doi: 10.1080/14756366.2016.1204610
